# Radiographic Templating for Tarsometatarsal Operative Fixation: A Retrospective Study

**DOI:** 10.7759/cureus.87375

**Published:** 2025-07-06

**Authors:** Adeeb Alomar, Dang-Huy Do, Trapper Lalli, Drew Sanders

**Affiliations:** 1 Orthopedic Surgery, University of Texas Southwestern Medical Center, Dallas, USA; 2 Orthopedic Surgery, University of North Carolina Medical Center, Chapel Hill, USA

**Keywords:** fixation, midfoot, radiograph, surgical, tarsometatarsal, template

## Abstract

Objective: For midfoot injuries requiring surgical intervention, radiographs of the contralateral, non-injured foot are often used as a guide to restore patient anatomy. We seek to validate this approach by examining the intra-subject variability in midfoot anatomy among the uninjured population.

Methods: A retrospective review of 440 patients with bilateral foot radiographs was performed. A total of 246 patients met the inclusion criteria. The first to second intermetatarsal angle (IMA), talo-first-metatarsal angle (T1MA), Meary’s angle (MA), and calcaneal inclination (CI) were measured. The side-to-side difference and inter-subject variability were analyzed using the mean absolute percentage side-to-side difference (MAPSSD) and the coefficient of variation (COV) to yield the ratio of variation (ROV). An ROV greater than 1 indicates greater inter-subject variability than intra-subject differences. Standard linear model analysis was performed to study each parameter against sex, race, and age.

Results: There were no significant differences in IMA, T1MA, or CI in side-by-side measurements, but there was a significant difference in MA between the left and the right side. The ROV for IMA, T1MA, MA, and CI were all greater than 1, indicating greater inter-subject variability than intra-subject variability between the left and the right sides. Male sex was associated with a smaller IMA. Hispanic heritage was associated with smaller T1MA. Older age and Black people were associated with smaller MA angles. Caucasian people had larger CI values. All correlations were statistically significant with a p-value < 0.05.

Conclusions: Intra-subject variability in midfoot anatomy is less than inter-subject variability, therefore validating the use of the contralateral limb as a guide in the surgical repair of midfoot injuries.

## Introduction

The tarsometatarsal complex is comprised of multiple small bone articulations that provide stability to the midfoot, maintaining both the longitudinal and transverse arches of the midfoot [1]. Tarsometatarsal complex injuries are a wide category of foot injuries ranging in severity from ligamentous sprains to high-energy, comminuted fracture patterns. Failure to identify and properly treat this injury can lead to permanent deformity, pain, arthritis, and loss of function, increasing patient morbidity. Management of tarsometatarsal complex injuries includes non-surgical options for non-displaced and stable injuries and operative options for displaced and unstable injuries. Kinner et al. showed that early patient outcomes of complex foot trauma management were determined by soft tissue injury, whilst the long-term patient outcomes were heavily determined by the severity of trauma to the bones and joints [2]. It has been shown that the one factor affecting functional outcomes after surgery is the quality of the reduction, with anatomical reduction being the primary aim [3].

Frequently, when treating displaced fractures and dislocations, the contralateral limb can be used as a template to guide the reconstruction of the injured site. It has been shown by Rogers et al. and Raji et al. that such an approach of using the contralateral structure as a guide is useful in managing fractures of the proximal femur as well as the calcaneus, respectively [4,5]. Surgeons continue to use a similar strategy in reconstructing tarsometatarsal joint injuries of the foot, especially for comminuted fractures. Restoring appropriate adduction/abduction and flexion/extension of the first ray is crucial for maintaining alignment with the overall tarsometatarsal complex. There are currently no known studies comparing the anatomic variability of the feet and to which this variation may be. This study examines the anatomic variability in patients using bilateral foot radiographs. The aim is to validate the use of contralateral, non-injured foot radiographs as a guide to restore patient anatomy in midfoot injuries requiring surgical intervention.

## Materials and methods

This is a retrospective cohort study of patients with bilateral foot radiographs. The study was approved by the University of Texas Southwestern Institutional Review Board (approval no. STU-2023-0228). An a priori power analysis was performed to calculate the appropriate sample size to achieve an α-value of 0.05 and a statistical power of 0.80. The necessary sample size was determined to be 125 patients. Additionally, prior publications evaluating bilateral calcaneal alignment used a sample size of 200 patients [5]. Our institution’s Philips iSite Picture Archiving Communication System (PACS) was used to query all patients with bilateral foot radiographs between October 2022 and October 2023.

The inclusion criteria for this study were patients with bilateral three-view foot radiographic films (anteroposterior, oblique, and lateral) for non-traumatic conditions and aged 18 to 89 years. Exclusion criteria included age less than 18 or over 89 years, presence of fractures on either foot, previous history of foot surgeries, radiographic evidence of arthrosis in ankle, hindfoot, or midfoot, osteomyelitis, Charcot arthropathy, neuropathy, tumor, hammertoes, and congenital foot deformities. Bilateral foot radiographs obtained in the setting of trauma screening in the emergency room or for foot pain in the clinic that did not have any of the aforementioned injuries or pathologies were included.

A review of the electronic medical records was performed to collect demographic information, including age, sex, and race/ethnicity, and screen for relevant medical and surgical history. Once films were confirmed to meet the criteria of this study, key anatomical angles, including the 1st intermetatarsal angle (IMA), the talo-first metatarsal angle (T1MA), Meary’s angle (MA), and calcaneal inclination (CI), were measured. Before initiation, 15% of the total films included in this study were measured independently by three raters to assess reproducibility (authors AA, DD, and DS). All radiographs were measured by author AA using the PACS software.

**Figure 1:**
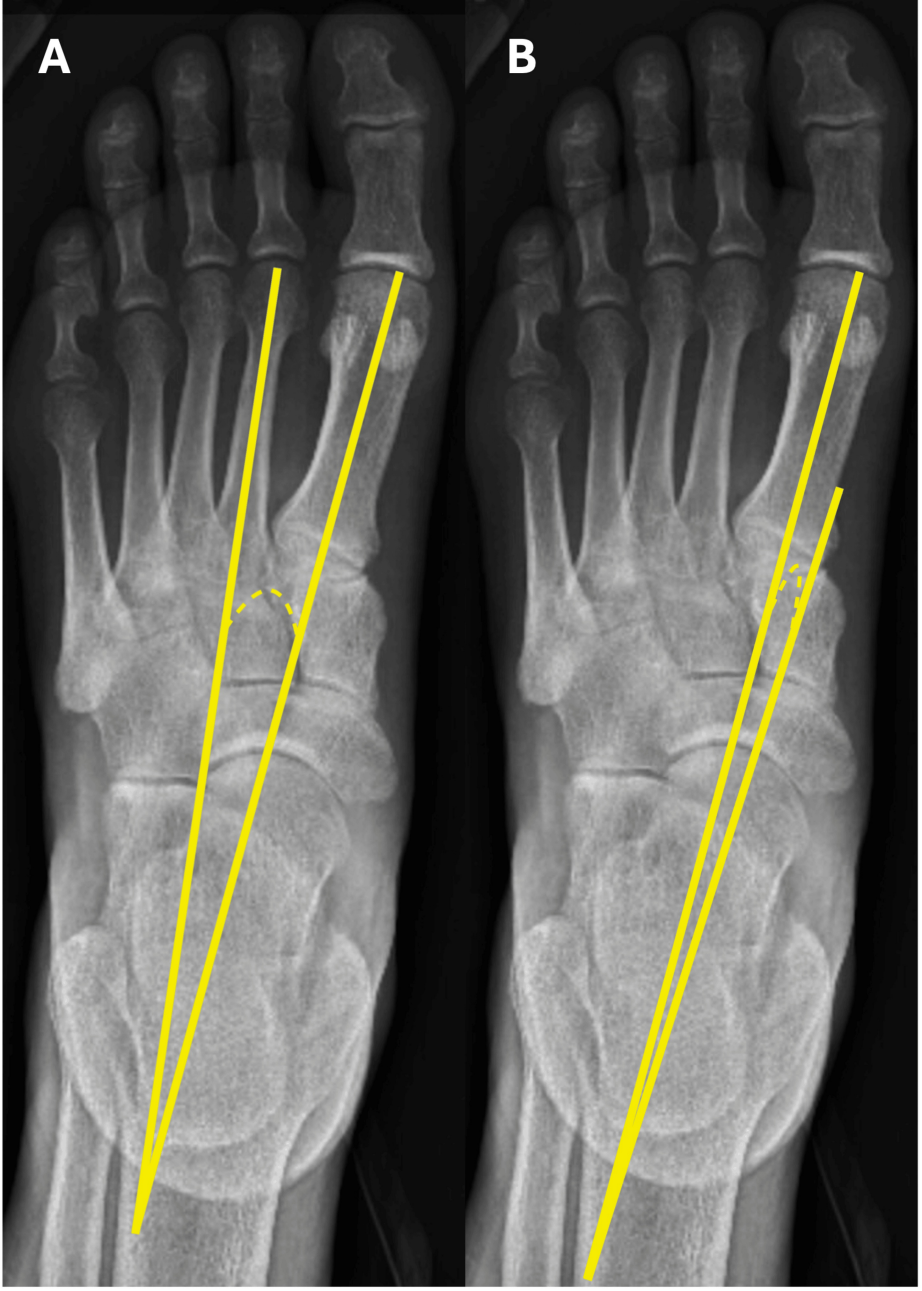
The IMA (A) and T1MA (B) A: The IMA is defined as the angle between the long axis of the first metatarsal bone and the long axis of the second metatarsal bone.; B: The T1MA is defined as the angle between the long axis of the talus and the long axis of the first metatarsal bone. IMA: Intermetatarsal angle, T1MA: Talo-first metatarsal angle

**Figure 2:**
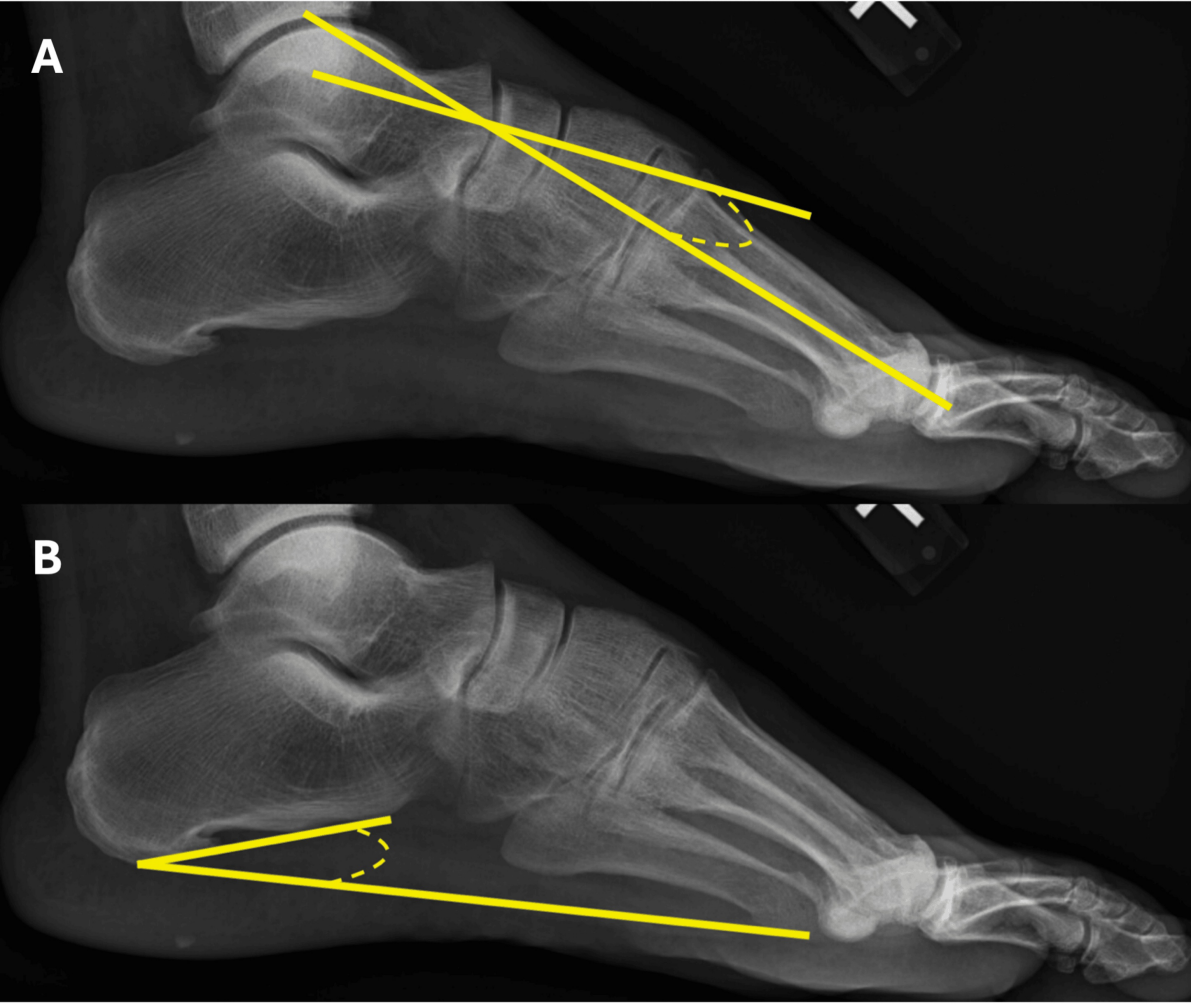
Meary's angle (A) and CI (B) A: The MA is defined as the angle between the long axis of the talus and the long axis of the first metatarsal bone; B: The CI is defined as the angle formed between a line drawn from the most inferior point of the calcaneal medial process to the inferior most point of the calcaneal-navicular articulation and a line drawn from the most inferior point of the calcaneal medial process to the head of the fifth metatarsal. MA: Meary's angle; CI: Calcaneal inclination

The IMA was defined as the angle between the long axis of the first metatarsal bone and the long axis of the second metatarsal bone (see Figure 1, panel A) [6]. The T1MA was defined as the angle between the long axis of the talus and the long axis of the first metatarsal bone (see Figure 1, panel B) [7]. The MA was defined as the angle between the long axis of the talus and the long axis of the first metatarsal bone (see Figure 2, panel C) [8]. The CI was defined as the angle formed between a line drawn from the most inferior point of the calcaneal medial process to the inferior most point of the calcaneal-navicular articulation and a line drawn from the most inferior point of the calcaneal medial process to the head of the fifth metatarsal (see Figure 2, panel D) [8]. The IMA and T1MA were measured on anterior-posterior radiographs, whereas MA and CI were measured on lateral films.

The dataset of this study consisted of left- and right-sided measurements of IMA, T1MA, MA, and CI, as well as the population demographics, including age, sex, and race/ethnicity. The primary goal of the analysis was to compare inter-subject variability in the selected anatomical parameters against intra-subject variability. An additional analysis was carried out to investigate the correlation between age, sex, race/ethnicity, and the selected angles. 

For each parameter, inter-subject variability was assessed using the coefficient of variation (COV), which is defined as the percentage of the standard deviation over the mean of the angle: \(COV=(\frac{\text{SD}}{\text{mean of angle}})\times 100)\. Intra-subject variability was assessed using the mean absolute percentage side-to-side difference (MAPSSD), defined as the percentage of the side-to-side difference over the side-to-side mean: \(MAPSSD=(\frac{\text{side-to-side difference}}{\text{side-to-side mean}})\times 100)\. A comparison between these two variables yields the ratio of variation (ROV), defined as the COV divided by the MAPSSD: \(ROV=\frac{COV}{MAPSSD})\.

A ratio greater than 1 indicates greater inter-subject variability compared to intra-subject variability. Additionally, the Mann-Whitney U test was used for side-to-side comparison of the measurements, given the nonparametric nature of the data. The calculation of the Mann-Whitney U test is shown in the equation \(U = n_{1}n_{2} + \frac{n_{2}(n_{2}+1)}{2}-R_{1})\. Here, "n1" is the number of observations in sample A (left side), "n2" is the number of observations in sample B (right side), and R1 is the sum of ranks of the observations in sample A. This is repeated for sample B to identify statistical differences between the two samples (i.e., the left side and the right side). 

To assess the reproducibility of the measurement, 15% of the total measurements for each parameter were completed independently by three raters (a medical student, a third-year orthopedic resident, and an orthopedic trauma attending), and results were analyzed using a two-way mixed effects intraclass correlation (ICC) analysis given the metric nature of the data. The ICC calculation is as follows: \(ICC=\frac{MS_{R}-MS_{E}}{MS_{R}+\frac{MS_{C}-MS_{E}}{n}})\. Here, "MSR" is mean square for rows, "MSE" is mean square for error, "MSC" is mean square for columns, and "n" is the number of subjects. This calculation was performed using a programmed code script in R (R Foundation for Statistical Computing, Vienna, AUT). 

The ICC values less than 0.50 were reported as poor reliability, 0.50-0.74 as moderate reliability, 0.75-0.89 as good reliability, and values greater than 0.90 as excellent reliability [9]. To examine the angle correlation with demographics, a standard linear regression model analysis was done. Additionally, a Spearman rank-order correlation was calculated to study the relationship between angle and age: \(rho=1-\frac{6 \sum d_{i}^{2}}{n (n^{2}-1)})\. Here, "di" is the difference between the two ranks of each observation, and "n" is the number of observations. All tests performed were two-sided, and a p-value < 0.05 was considered statistically significant. 

## Results

Bilateral foot radiographs for 440 patients were reviewed for this study. Of these, 246 patients met the inclusion criteria and were included in the analysis. The study included 147 females (60%) and 99 males (40%) with a mean age of 53.0 ± 13.5 years. The study population patients were 46% Hispanic, 32% Black, 17% Caucasian, 3% Asian, and 2% other races/ethnicities (Table 1). 

**Table 1:** Study demographics

Variables	Categories	Percentage (n); Total n = 246
Gender	Female	60% (147)
	Male	40% (99)
Race/ethnicity	Hispanic	46.3% (114)
	Black	32.1% (79)
	Caucasian	17.1% (42)
	Asian	3.3% (8)
	Other	1.2% (3)

The mean IMA was 8.9° ± 3.4° for the left side and 9.3° ± 3.4° for the right side. There was no significant relationship between IMA and race/ethnicity or age. The IMA was smaller in males (8.0° ± 2.6°) compared to females (9.8° ± 3.1°) (p < 0.001). The mean T1MA was 5.3° ± 5.0° for the left side and 5.8° ± 6.0° for the right side. There was no significant relationship between T1MA and sex or age. The T1MA was lower in Hispanic patients (4.8° ± 3.8°) compared to Caucasian patients (6.8° ± 5.1°) (p = 0.011). The mean MA was 10.5° ± 9.3° for the left side and 8.6° ± 9.5° for the right side. There was no significant relationship between MA and sex, but MA was lower in Black patients (7.9° ± 8.9°) compared to Caucasian patients (12.1° ± 6.8°) (p = 0.019). Additionally, MA was affected by age such that increased age was associated with smaller MA values (p = 0.003). The mean CI was 20.1° ± 6.5° for the left side and 20.2° ± 6.8° for the right side. There was no significant relationship between CI and sex or age, but CI was larger in Caucasian patients (23.4° ± 5.0°) compared to non-Caucasian patients (19.5° ± 6.4°) (p < 0.001).

Of the subjects that were selected randomly, ICC analysis ranged between 0.57 and 0.91, with an average ICC value of 0.77 for all measurements. There was good to excellent agreement among the three independent observers over IMA measurements (0.86-0.91). Agreement ranged between moderate and good for CI (0.68-0.72) and T1MA (0.57-0.75), while MA had good agreement (0.78-0.88). All ICC analyses were followed by an analysis of variance (ANOVA) test, and results were statistically significant with p < 0.001 (Table 2).

**Table 2:** Intraclass correlation analysis for inter-rater agreement *A p < 0.05 is considered statistically significant. The ICC values less than 0.50 were reported as poor reliability, 0.50-0.74 as moderate reliability, 0.75-0.89 as good reliability, and values greater than 0.90 as excellent reliability. The p-values were calculated using ANOVA. ICC: Intraclass correlation; ANOVA: Analysis of variance; IMA: Intermetatarsal angle; T1MA: Tntermetatarsal angle; MA: Meary’s angle; CI: Calcaneal inclination

Parameter	ICC value	Reliability	p-value
Left IMA	0.86	Good	<0.001*
Right IMA	0.91	Excellent	<0.001*
Left T1MA	0.57	Moderate	<0.001*
Right T1MA	0.75	Good	<0.001*
Left MA	0.78	Good	<0.001*
Right MA	0.88	Good	<0.001*
Left CI	0.72	Moderate	<0.001*
Right CI	0.68	Moderate	<0.001*

A Spearman correlation test was used to study the relationship between age and each parameter in the study. A statistically significant relationship was found between MA and age. The MA decreases with age (p < 0.001). Age did not have a significant relationship with IMA, T1MA, or CI (Table 3).

**Table 3:** Spearman’s rank-order correlation coefficients between age and angle *A p < 0.05 is considered statistically significant. The correlation coefficient "r" ranges from -1 to +1 and represents the strength and direction of the linear relationship between the angle and age. The p-values were calculated using the Spearman rank-order correlation test. IMA: Intermetatarsal angle; T1MA: Tntermetatarsal angle; MA: Meary’s angle; CI: Calcaneal inclination

Measurement	Left side		Right side		Mean	
	r	p-value	r	p-value	r	p-value
IMA (degrees)	0.09	0.16	0.01	0.93	0.07	0.29
T1MA (degrees)	0.03	0.61	0.00	0.99	0.00	0.96
MA (degrees)	-0.22	<0.001*	-0.19	0.003*	-0.22	<0.001*
CI (degrees)	-0.09	0.16	-0.03	0.69	-0.07	0.29

Finally, there were no significant differences in IMA, T1MA, or CI in comparing the left side against the right side. However, there was a statistically significant difference in MA between the left and the right side (p = 0.015). The ROV for IMA, T1MA, MA, and CI were all > 1, indicating a greater inter-subject variability than intra-subject variability between the left and the right side. For IMA, the MAPSSD was 24.6% and COV was 37.3, yielding an ROV of 1.5. For T1MA, the MAPSSD was 82.3% and COV was 99.2, yielding an ROV of 1.2. For MA, the MAPSSD was 75.5% and COV was 99.1, yielding an ROV of 1.3. For CI, the MAPSSD was 15.4% and COV was 33.0, yielding an ROV of 2.2 (Table 4).

**Table 4:** Inter-subject and intra-subject variability of metatarsal parameters *A p < 0.05 is considered statistically significant. The p-values were calculated using the Mann-Whitney U test. IMA: Intermetatarsal angle; T1MA: Tntermetatarsal angle; MA: Meary’s angle; CI: Calcaneal inclination

Parameter	Left side (mean ± SD)	Right side (mean ± SD)	Mean absolute percentage of side-to-side difference (mean ± SD)	Coefficient of variation	Ratio of variation	p-value
IMA	8.9° ± 3.4°	9.3° ± 3.4°	24.6 ± 21.9	37.3	1.5	0.17
T1MA	5.3° ± 5.0°	5.8° ± 6.0°	82.6 ± 60.2	99.2	1.2	0.71
MA	10.5° ± 9.3°	8.6° ± 9.5°	75.5 ± 110.3	99.1	1.3	0.02*
CI	20.1° ± 6.5°	20.2° ± 6.8°	15.4 ± 15.6	33.0	2.2	0.84

## Discussion

Surgical correction of injuries to the tarsometatarsal complex is often performed with open reduction with direct visualization of the joint, but there are many scenarios where restoration may still be challenging. For example, for extensively comminuted base fractures, the joint surface may not be intact to guide reduction and alignment. Additionally, because the first tarsometatarsal joint is dome-shaped, the surgeon can easily introduce adduction or abduction deviations of the first ray, leading to hallux varus or valgus, respectively, or flexion or extension, leading to cavus or planus, respectively. Therefore, reliance on the contralateral limb as a template is valuable. This study aims to validate this approach in repairing injuries of the tarsometatarsal complex by investigating the intra-subject variability between the left and right sides and comparing it against inter-subject variability to determine whether the contralateral limb can be used as an anatomical guide. Additionally, this study examines the relationship between key anatomical parameters of the midfoot and subject characteristics such as age, sex, and race/ethnicity. 

Using the contralateral limb as an anatomical guide in surgical reduction is a common strategy in addressing injuries of various joints in the body. Rogers et al. performed a retrospective cohort study to investigate side-to-side variability of the femoral neck shaft angle (NSA) using anteroposterior (AP) pelvis radiographs [4]. Their analysis found no significant variability between the left and right side NSAs [4]. Raji et al. performed a similar study to evaluate side-to-side variability in calcaneal parameters such as Böhler angle (BA), crucial angle of Gissane (CAG), calcaneal length (CL), calcaneal height (CH), and calcaneotalar ratio (CTR) [5]. They also evaluated inter-subject variability of these parameters and concluded that intra-subject variability was smaller than inter-subject variability [5].

In this retrospective study, the inter-subject variability was assessed using the COV calculated for each parameter from the sample population. Intra-subject variability was assessed using the MAPSSD. A comparison of these two variables was done to yield ROV. A ratio >1 would indicate that inter-subject variability in a parameter is less than the side-to-side difference in the selected parameter. The ROV was >1 in all four parameters studied in this analysis. This indicates the contralateral midfoot can be used as a reliable guide of anatomy. 

Additionally, IMA was affected by sex, i.e., males had a smaller IMA, whereas T1MA was affected by race/ethnicity: Hispanic patients were associated with smaller T1MA values. Moreover, MA was affected by race/ethnicity and age; Black patients and increased age were associated with smaller MA values. Interestingly, CI demonstrated a strong relationship with race/ethnicity, such that Caucasian patients were associated with a larger CI angle compared to any other race/ethnicity. These findings build on correlations of the foot anatomy with patient characteristics found by Raji et al. in their calcaneal study. In their analysis, Raji et al. concluded that the female sex was associated with smaller BA, smaller CL, and smaller CH, whereas increased age was associated with larger CAG and smaller CTR [5]. These findings help better understand how the anatomy of the foot is affected by various patient characteristics, thereby providing the surgeon with valuable knowledge to consider in evaluating foot deformities in adults. Furthermore, these findings highlight that Black and Hispanic patients are associated with smaller CI values. As CI provides an estimate of the arch of the foot, these findings are consistent with previous studies that found African American patients to be three times more likely to have pes planus compared to Caucasian patients [10,11].

This study is not without limitations. There was a statistically significant difference in MA in the overall left and right side comparison. Varying degrees of asymmetry between feet have been established, and differences in the arches of an individual’s feet are not uncommon, which could explain the difference in MA. While a template is never perfect, when attempting to restore an injured foot to a more normal position, the surgeon may benefit from subtle cues of alignment derived from the patient’s opposite foot. Additionally, we did not correlate the differences in angles with clinical outcomes, which is beyond the scope of this study. The strength of this study is that double the necessary sample size was obtained from the a priori power analysis, which also exceeds the sample size of similar studies in the literature. Another important strength of this study is the relatively large sample size and the high statistical power of the correlations identified during the statistical analysis. 

## Conclusions

This is a retrospective analysis of key anatomical parameters of the midfoot to investigate side-to-side differences and compare them against inter-subject variability. Race/ethnicity was found to have a strong effect on prominent markers of the arch of the midfoot. Additionally, there is strong evidence that intra-subject variability in the anatomy of the midfoot is less variable among patients, validating the use of the contralateral limb as a guide to achieve anatomical reduction in surgical repair of injuries to the midfoot.
